# Web alert: Engineered carbon dioxide capture: An annotated selection of World Wide Web sites relevant to the topics in microbial biotechnology

**DOI:** 10.1111/1751-7915.14202

**Published:** 2022-12-30

**Authors:** Lawrence P. Wackett

**Affiliations:** ^1^ Department of Biochemistry, Molecular Biology & Biophysics, BioTechnology Institute University of Minnesota St. Paul Minnesota USA

## CO_2_ fixation and conversion to products

### https://www.sciencedirect.com/science/article/pii/S1385894720305751

This article reviews microbial carbon dioxide fixation for the purpose of making useful chemicals from a chemical engineering perspective.

## Developing artificial autotrophs

### https://www.frontiersin.org/articles/10.3389/fmicb.2020.592631/full

Natural organisms have evolved to fix carbon dioxide for their survival purposes, and producing artificial autotrophic organisms might provide for more beneficial microbes as cell factories for biotechnology.

## CO_2_ fixation for microbial biotechnology

### https://academic.oup.com/femsle/pages/microbial_carbon_dioxide_fixation

This link is to a thematic issue compendium dealing with CO_2_ fixation in biotechnology published in *FEMS Microbiology Letters*. The issue includes studies of naturally occurring bacteria and engineered enzymes.

## Carboxysomes

### https://www.cell.com/trends/microbiology/fulltext/S0966‐842X(21)00259‐6

This review article deals with recent advances in studies of microbial organelles for CO_2_ fixation.

## Microbial CO_2_ fixation in soils

### https://onlinelibrary.wiley.com/doi/full/10.1111/gcb.14937

This article discusses largely unknown sources of carbon dioxide fixation, sometimes denoted as microbial CO_2_ fixation.

## Heterotrophic contribution to carbon cycling

### https://bg.copernicus.org/preprints/bg‐2020‐465/bg‐2020‐465.pdf

Heterotrophs catalyse many different carboxylation reactions. This has often been ignored as a contribution to large‐scale carbon dioxide fixation, but the report suggests it is significant.

## Extracellular electron uptake and CO_2_ fixation

### https://www.nature.com/articles/s41467‐019‐09377‐6

Here, it is discussed that electrons from solid‐phase conductive surfaces can stimulate photosynthetic carbon dioxide fixation.

## Rapid reductive carboxylation

### https://pubs.acs.org/doi/10.1021/acscentsci.2c00057

This report investigates enoyl‐CoA carboxylases/reductases as some of the most efficient CO_2_ fixing enzymes described to date.

## Designing CO_2_ fixation

### https://www.ibiology.org/bioengineering/synthetic‐carbon‐dioxide‐fixation/

This link takes viewers to an interesting video on synthetic carbon dioxide fixation.

## Biological and chemical strategies for CO_2_ fixation

### https://pdfs.semanticscholar.org/5603/bf718d33bea7bdc07fc9877529b79c498934.pdf

There are many efforts to sequester carbon dioxide from the atmosphere. This report seeks to be comprehensive and approaches the topic from a range of synthetic chemical and biological perspectives.

## CO_2_ fixation by engineered Escherichia coli

### https://elifesciences.org/articles/59882

This engineering of CO_2_ fixation serves to provide information of adaption for an autotrophic lifestyle.

## Rhodopsin and CO_2_ fixation

### https://journals.plos.org/plosone/article?id=10.1371/journal.pone.0246656

This report is a metagenomic study focussed on carbon dioxide fixation and rhodopsin proteins in microbes found in mats in hypersaline lakes.

## Revving up artificial photosynthesis

### https://www.sciencedaily.com/releases/2022/04/220429185744.htm

This is a popular science article describing high‐flux carbon dioxide fixation by a modified microbial enzyme.

